# Machine Learning–Based Prediction of Delirium and Risk Factor Identification in Intensive Care Unit Patients With Burns: Retrospective Observational Study

**DOI:** 10.2196/65190

**Published:** 2025-03-05

**Authors:** Ryo Esumi, Hiroki Funao, Eiji Kawamoto, Ryota Sakamoto, Asami Ito-Masui, Fumito Okuno, Toru Shinkai, Atsuya Hane, Kaoru Ikejiri, Yuichi Akama, Arong Gaowa, Eun Jeong Park, Ryo Momosaki, Ryuji Kaku, Motomu Shimaoka

**Affiliations:** 1 Department of Molecular Pathobiology and Cell Adhesion Biology, Mie University Graduate School of Medicine Mie University Tsu Japan; 2 Department of Practical Nursing Mie University Graduate School of Medicine Tsu city Japan; 3 Department of Medical Informatics Mie University Hospital Tsu city Japan; 4 Department of Emergency and Critical Care Center Mie University Hospital Tsu city Japan; 5 Department of Rehabilitation Medicine Mie University Graduate School of Medicine Tsu city Japan; 6 Department of Anesthesiology Mie University Hospital Tsu city Japan

**Keywords:** burns, delirium, intensive care unit, machine learning, prediction model, artificial intelligence, AI

## Abstract

**Background:**

The incidence of delirium in patients with burns receiving treatment in the intensive care unit (ICU) is high, reaching up to 77%, and has been associated with increased mortality rates. Therefore, early identification of patients at high risk of delirium onset is essential for improving treatment strategies.

**Objective:**

This study aimed to create a machine learning model for predicting delirium in patients with burns during their ICU stay using patient data from the first day of ICU admission and identify predictive factors for ICU delirium in patients with burns.

**Methods:**

This study focused on 82 patients with burns aged ≥18 years who were admitted to the ICU at Mie University Hospital for ≥24 hours between January 2015 and June 2023. In total, 70 variables were measured in patients upon ICU admission and used as explanatory variables in the ICU delirium prediction model. Delirium was assessed using the Intensive Care Delirium Screening Checklist every 8 hours after ICU admission. A total of 10 different machine learning methods were used to predict ICU delirium. Multiple receiver operating characteristic curves were plotted for various machine learning models, and the area under the curve (AUC) for each was compared. In addition, the top 15 risk factors contributing to delirium onset were identified using Shapley additive explanations analysis.

**Results:**

Among the 10 machine learning models tested, logistic regression (mean AUC 0.906, SD 0.073), support vector machine (mean AUC 0.897, SD 0.056), k-nearest neighbor (mean AUC 0.894, SD 0.060), neural network (mean AUC 0.857, SD 0.058), random forest (mean AUC 0.850, SD 0.074), adaptive boosting (mean AUC 0.832, SD 0.094), gradient boosting machine (mean AUC 0.821, SD 0.074), and naïve Bayes (mean AUC 0.827, SD 0.095) demonstrated the highest accuracy in predicting ICU delirium. Specifically, 24-hour urine output (from ICU admission to 24 hours), oxygen saturation, burn area, total bilirubin level, and intubation upon ICU admission were identified as the major risk factors for delirium onset. In addition, variables, such as the proportion of white blood cell fractions, including monocytes; methemoglobin concentration; and respiratory rate, were identified as important risk factors for ICU delirium.

**Conclusions:**

This study demonstrated the ability of machine learning models trained using vital signs and blood data upon ICU admission to predict delirium in patients with burns during their ICU stay.

## Introduction

### Background

Delirium is a significant complication in patients in the intensive care unit (ICU) and is recognized as an urgent medical need requiring treatment and prevention. Delirium is defined as acute brain dysfunction associated with underlying conditions characterized by fluctuating bouts of impaired consciousness, attention, and cognition. This condition is frequently observed in patients admitted to the ICU, with delirium occurring in 10% to 50% of patients [1]. Delirium is an independent predictor of poor outcomes, and there is currently no established specific treatment, making early diagnosis and prevention critically important [2]. In particular, the incidence of delirium in patients with burns can reach 77% [3], with reports indicating that 30% of patients who develop delirium respond effectively to prevention and treatment [4]. These facts underscore the importance of identifying patients at high risk of delirium and implementing preventive measures.

In recent years, research on ICU delirium prediction using artificial intelligence technology has advanced, with a particular focus on the application of various machine learning algorithms. These algorithms, such as random forest (RF), support vector machine (SVM), and gradient boosting, can be used to develop predictive models for ICU delirium [5]. While these algorithms themselves do not identify relevant features, they can be combined with feature importance analysis techniques such as Shapley additive explanations (SHAP) to determine which variables contribute most significantly to the predictions. This approach allows for the analysis of large volumes of patient data with speed and accuracy beyond human capacity. However, research on the prediction of ICU delirium in patients with burns remains underdeveloped, and very few studies have been conducted in this field.

### Objectives

This study aimed to demonstrate whether it is possible to predict ICU delirium in patients with burns using machine learning. Specifically, we hypothesized that a machine learning model using clinical data such as vital signs and blood test results could predict delirium in ICU patients. The null hypothesis was that these models would not be superior to random chance in predicting ICU delirium.

While ICU delirium prediction potential for multiple machine learning models, as well as the model with the highest accuracy, was assessed, the second objective of this study was to identify risk factors for ICU delirium in patients with burns and contribute to the development of more effective prevention and treatment strategies.

This ICU delirium prediction approach using machine learning has the potential to support the early detection of ICU delirium in patients with burns and, ultimately, improve patient outcomes.

## Methods

### Patient Demographics and Data Collection

This was a retrospective observational study focused on predicting delirium in patients with burns admitted to the ICU. This study included 82 patients with burns aged ≥18 years who were admitted to the Mie University Hospital ICU for ≥24 hours between January 2015 and June 2023. The sample size of 82 patients was determined by including all patients with burns who were admitted to the ICU within the study period.

Patients were retrospectively included based on the inclusion criteria, which ensured the comprehensive capture of all eligible cases during the study period. This approach minimized selection bias and allowed for a representative sample of the population of patients with burns in our ICU.

Physiological, biochemical, and clinical data collected from these patients upon ICU admission were used to extract 70 explanatory variables. This study aimed to develop a model using these 70 variables to predict delirium onset during ICU admission, assess its accuracy, and identify the risk factors contributing to each model.

### The Definition, Diagnostic Criteria, and Standard Assessment of ICU Delirium

ICU delirium, also known as ICU psychosis, is an acute, fluctuating change in consciousness and cognition that occurs frequently in patients who are critically ill. ICU delirium is characterized by disturbances in attention, awareness, and cognitive function. These disturbances are often temporary and reversible but can lead to prolonged ICU stays, increased morbidity and mortality, and long-term cognitive impairments if not properly managed.

The standard assessment of ICU delirium involves the use of validated diagnostic tools to ensure accurate detection and timely intervention. In total, 2 widely recognized tools are the Confusion Assessment Method for the Intensive Care Unit (CAM-ICU) and the Intensive Care Delirium Screening Checklist (ICDSC).

The CAM-ICU is a structured diagnostic tool specifically designed for use in the ICU setting. It is based on the Confusion Assessment Method and has been modified for the critical care environment. The CAM-ICU assesses four key features: (1) acute onset of mental status changes or a fluctuating course, (2) inattention, (3) disorganized thinking, and (4) altered level of consciousness. A positive CAM-ICU diagnosis requires the presence of both features 1 and 2 and either feature 3 or 4. This tool is favored for its ease of use and quick administration, making it suitable for frequent assessments.

The ICDSC is another tool used to screen for delirium in ICU patients, consisting of an 8-item checklist that assesses various cognitive and behavioral symptoms associated with delirium. The items are (1) altered level of consciousness, (2) inattention, (3) disorientation, (4) hallucinations or delusions, (5) psychomotor agitation or retardation, (6) inappropriate speech or mood, (7) sleep and wake cycle disturbances, and (8) symptom fluctuation. Each item is scored based on its presence within the previous 24 hours, and a total score of ≥4 indicates the presence of delirium. The ICDSC provides a comprehensive overview of the patient’s condition over a longer period than the CAM-ICU.

In this study, delirium was assessed using the internationally recognized ICDSC every 8 hours after ICU admission. Delirium was diagnosed when the ICDSC score was ≥4 points. We opted for a binary classification approach because this method simplifies the model’s output to either *delirium present* or *delirium absent*, facilitating smoother decision-making in clinical settings. Another reason for this choice was the diversity in patient conditions in real-world clinical environments, where a few specific atypical cases can skew the predictions of regression models. Binary classification is less susceptible to the influence of such outliers, enabling the development of a more robust model. This method allowed for accurate determination of the presence and severity of delirium.

### Development of Machine Learning Models

In total, 10 different algorithms were used to develop the machine learning models: logistic regression (LR), RF, SVM, neural network, k-nearest neighbor (KNN), decision tree, naïve Bayes, adaptive boosting (AdaBoost), gradient-boosting machine (GBM), and linear discriminant analysis (LDA). These models were selected based on the area under the receiver operating characteristic curve (AUC) to compare the accuracy of delirium prediction.

### Ethical Considerations

This study was approved by the ethics committee of Mie University (H2020-164), ensuring compliance with ethical guidelines for clinical research. This study underwent ethical review and was approved as a clinical research study. For informed consent, we applied the opt-out method, allowing participants to refuse the inclusion of their data in this study. If participants chose to opt out, their data were excluded from the analysis. To protect privacy and confidentiality, all study data were fully anonymized. Data extracted from electronic medical records were deidentified, ensuring that individual patients could not be identified. No compensation was provided to participants. As the study used an opt-out approach, participants had the right to refuse the inclusion of their data without any consequences.

### Feature Importance Analysis Using SHAP

In this study, we used SHAP to interpret the feature importance of our machine learning models. SHAP provides a unified measure of each variable’s contribution to the prediction outcome. By applying SHAP, we identified the top 15 predictors of ICU delirium across all models, which allowed for a detailed understanding of the factors most associated with delirium onset. The SHA*P* values helped clarify the influence of variables such as 24-hour urine output, total bilirubin (T-bil) levels, and respiratory rate on the predictive performance of the models. SHAP analysis also enabled clinicians to visualize and interpret the risk factors, enhancing the practical applicability of the model’s predictions.

### Data Preprocessing for Machine Learning

#### Handling Missing Values

In this study, missing values were not handled. This approach ensured that all features had complete data, thus preserving the dataset’s integrity without excluding any records.

#### Data Splitting

The dataset was loaded from a CSV file using the *Pandas* library. It was then split into explanatory variables and the target variable, where the target variable was delirium. The data were divided into 2 subsets: 80% was allocated for training the model, and 20% was reserved for internal validation. To evaluate the model’s performance, the dataset was further split into folds using stratified k-fold cross-validation, which ensures that the proportion of classes remains consistent across folds.

#### Data Standardization

The explanatory variables in both the training and test sets were standardized using *StandardScaler* from the *sklearn* library to ensure that each feature had a mean of 0 and an SD of 1. This preprocessing step is crucial for models sensitive to feature scaling.

#### Model Selection and Initialization

Several machine learning models were initialized for this study, including SVM, neural network, KNN, decision tree, naïve Bayes, AdaBoost, GBM, LDA, LR, and RF. Each model was imported from the *sklearn* or *SciPy* library and initialized with appropriate default parameters.

#### Hyperparameter Tuning

For specific models, such as SVM, neural network, KNN, decision tree, and RF, hyperparameter tuning was performed using grid search with cross-validation. The *GridSearchCV* class from the *sklearn* library was used to search for the best combination of hyperparameters, optimizing the model’s performance based on the AUC metric.

#### Model Training and Evaluation

Each model was trained using stratified k-fold cross-validation to ensure robust performance evaluation. For models with hyperparameter grids defined, grid search was applied to find the best hyperparameter set. The best-performing model from the grid search was then used to fit the data in each fold. The model’s performance was evaluated using the AUC score on the test subset within each fold.

### Statistical Analysis

Continuous variables are reported as medians with IQRs and were compared using the Mann-Whitney *U* test or the Kruskal-Wallis test, depending on the number of groups. Categorical variables are expressed as counts and percentages, with comparisons made using the Fisher exact test or the chi-square test, as appropriate. All statistical analyses were conducted using the SPSS software (version 21; IBM Corp), and statistical significance was defined as *P*<.05.

To evaluate differences in model performance, pairwise 2-tailed *t* tests were applied to the AUC scores using the *ttest_ind* function from the *scipy.stats* module. In addition, a 1-way ANOVA was conducted using the *f_oneway* function to identify statistically significant differences across models.

Model performance results were visualized through bar charts displaying the mean AUC scores along with their SDs for each model. These visualizations were created using the *Matplotlib* library. A heat map was also generated to illustrate pairwise *P* values from the *t* tests, offering a clear representation of the statistical significance of the differences between models. The ANOVA *P* value was similarly calculated and displayed.

## Results

### Comparative Analysis of ICU Delirium in Patients With Burns

Compared to patients without delirium (Table 1), patients with burns with ICU delirium were older (age: median 77.0, IQR 69.5-84.5 years vs median 60.5, IQR 37.5-73.0 years; *P*<.001), were more likely to have airway burns (12/32, 38% vs 8/50, 16%; *P*=.03), and experienced longer ICU stays (median 2.5, IQR 1.8-11.2 days vs 2.0, IQR 1.0-3.0 days; *P*=.009). The mortality rate was significantly higher in the delirium group (7/32, 22% vs 0%; *P*=.001), and these patients more frequently required intubation (20/32, 62% vs 4/50, 8%; *P*<.001). The delirium group also had greater burn areas (median 16%, IQR 9.75%-34% vs 8%, IQR 4.6%-17%; *P*=.007) and burn indexes (median 9.5, IQR 4.7-25.1 vs median 4.0, IQR 1.0-10.0; *P*=.002).

**Table 1:** Characteristics of patients with burns with intensive care unit (ICU) delirium—comparison of explanatory factors between patients with and without deliriuma.

	Burns with delirium (n=32)	Burns without delirium (n=50)	*P* value
Age (y), median (IQR)	77.0 (69.5-84.5)	60.5 (37.5-73.0)	*<.001* ^b^
Sex (male), n (%)	20 (63)	32 (64)	.90
Height (cm), median (IQR)	160.5 (150.0-170.0)	162.2 (158.0-168.0)	.42
Weight (kg), median (IQR)	54.4 (44.4-63.3)	60.2 (50.5-67.1)	.07
BMI (kg/m^2^), median (IQR)	21.2 (19.0-23.4)	23.1 (19.7-24.9)	.06
Airway burns, n (%)	12 (38)	8 (16)	*.03* ^b^
Length of ICU stay (days), median (IQR)	2.5 (1.8-11.2)	2.0 (1.0-3.0)	*.009* ^b^
Mortality rate, n (%)	7 (22)	0 (0)	*.001* ^b^
Intubation, n (%)	20 (62)	4 (8)	*<.001* ^b^
Burn area (%), median (IQR)	16 (9.75-34)	8 (4.6-17)	*.007* ^b^
Burn index, median (IQR)	9.5 (4.7-25.1)	4.0 (1.0-10.0)	*.002* ^b^
WBC^c^ (10^3^ per μL), median (IQR)	11.9 (9.0-18.5)	8.8 (6.5-11.3)	*.006* ^b^
RBC^d^ (10^6^ per μL), median (IQR)	4.3 (3.7-5.3)	4.4 (4.0-4.9)	.51
Hemoglobin (g/dL), median (IQR)	13.5 (11.9-15.9)	14.6 (14.0-15.9)	*.04* ^b^
Hematocrits (%), median (IQR)	40 (35-44)	43 (41-46.7)	*.009* ^b^
MCV^e^ (fL), median (IQR)	92.0 (89.9-93.8)	92.3 (87.7-95.6)	.95
MCH^f^ (pg), median (IQR)	30.8 (30.0-32.4)	31.4 (29.7-32.9)	.71
MCHC^g^ (%), median (IQR)	33.8 (33.2-34.6)	33.9 (33-34.6)	.77
Platelet count (10^3^ per μL), median (IQR)	205.5 (165.0-314.8)	249.5 (202.2-302.0)	.50
Neutrophils (%), median (IQR)	77 (73.9-83.7)	74.4 (64.6-80.9)	*.04* ^b^
Lymphocytes (%), median (IQR)	15.8 (8.4-17.9)	16.8 (12.6-23.6)	*.02* ^b^
Monocytes (%), median (IQR)	6.6 (5.2-7.7)	6.6 (5.7-7.2)	.73
Eosinophils (%), median (IQR)	0.6 (0.4-1.4)	1.2 (0.6-1.9)	*.04* ^b^
Basophils (%), median (IQR)	0.4 (0.2-0.5)	0.4 (0.2-0.5)	.84
Neutrophil count (10^3^ per μL), median (IQR)	8985 (6265-10,830)	6840.0 (4449.2-9029.0)	*.03* ^b^
Lymphocyte count (10^3^ per μL), median (IQR)	1550 (1130-1943)	1550.0 (1178.2-2145.0)	.55
Monocyte count (10^3^ per μL), median (IQR)	767 (478-993)	575.0 (447.5-767.0)	*.04* ^b^
Eosinophil count (10^3^ per μL), median (IQR)	70.0 (37.5-111.0)	90.0 (40.0-167.7)	.22
Basophil count (10^3^ per μL), median (IQR)	38.5 (30.0-62.5)	37.0 (20.0-50.0)	.13
APTT^h^ (seconds), median (IQR)	30.8 (25.9-36.8)	27.4 (24.9-29.9)	*.02* ^b^
PT^i^ (seconds), median (IQR)	12.2 (11.5-13.6)	11.3 (10.8-12.1)	*.001* ^b^
PT (%)^j^, median (IQR)	94.8 (77-104.1)	105.4 (97-116.9)	*.001* ^b^
PT-INR^k^, median (IQR)	1.0 (0.9-1.1)	0.9 (0.9-1.0)	*.002* ^b^
Fibrinogen (mg/dL), median (IQR)	299.0 (251.2-374.2)	272.5 (225.2-327.7)	.20
D-dimer (μg/mL), median (IQR)	4.0 (1.5-8.5)	1.0 (0.2-8.2)	*.02* ^b^
pH, median (IQR)	7.3 (7.3-7.4)	7.4 (7.3-7.4)	*.04* ^b^
PCO_2_^l^ (mm Hg), median (IQR)	40.0 (31.7-45.0)	36.5 (33.2-39.7)	.28
PO_2_ (mm Hg), median (IQR)	152.5 (93.5-322.7)	123.0 (83.2-166.0)	*.03* ^b^
SO_2_^m^ (%), median (IQR)	99.1 (98.3-99.6)	97.6 (94.9-99)	*.01* ^b^
HCO_3_−^n^ (mmol/L), median (IQR)	23.1 (18.9-25.6)	23.1 (20.5-24.4)	.76
Anion gap (mmol/L), median (IQR)	10.4 (4.4-17.9)	13.1 (10.4-17.2)	.24
O_2_-Hb^o^ (%), median (IQR)	96.0 (92.5-97.3)	95.9 (90.8-97.1)	.59
CO-Hb^p^ (%), median (IQR)	1.6 (1.1-3.4)	1.7 (0.7-3.4)	.75
MetHb^q^ (%), median (IQR)	0.9 (0.5-1.1)	0.4 (0.3-0.9)	*.004* ^b^
Ionized calcium (mmol/L), median (IQR)	1.1 (1.0-1.1)	1.1 (1.0-1.1)	.60
Lactate (mmol/L), median (IQR)	3.0 (1.5-4.9)	2.3 (1.8-3.3)	.45
TP^r^ (g/dL), median (IQR)	6.4 (5.9-6.7)	7.0 (6.2-7.5)	*.006* ^b^
Albumin (g/dL), median (IQR)	3.6 (3.1-3.9)	4.1 (3.2-4.4)	*.004* ^b^
BUN^s^ (mg/dL), median (IQR)	16.9 (12.8-22.7)	13.4 (11.0-19.0)	.01
Creatinine (mg/dL), median (IQR)	0.8 (0.6-1.0)	0.7 (0.5-0.8)	*.03* ^b^
eGFR^t^ (mL per minute per 1.73 m^2^), median (IQR)	67.2 (47.8-87.2)	83.7 (59.9-101.0)	*.02* ^b^
Sodium (mmol/L), median (IQR)	139.0 (138.0-141.0)	140.0 (138.0-141.0)	>.99
Potassium (mmol/L), median (IQR)	4.0 (3.7-4.5)	3.9 (3.7-4.4)	.58
CL^u^ (mmol/L), median (IQR)	105.0 (102.0-107.0)	104.0 (103.0-106.7)	.57
Calcium (mg/dL), median (IQR)	8.5 (8.2-8.9)	8.9 (8.4-9.2)	*.009* ^b^
AST^v^ (U/L), median (IQR)	39.5 (22.7-60.5)	30.5 (24.2-38.0)	.30
ALT^w^ (U/L), median (IQR)	23.0 (14.0-43.5)	20.5 (14.5-29.0)	.46
LDH^x^ (U/L), median (IQR)	294.5 (216.7-630.7)	255.5 (197.7-303.0)	.05
ALP^y^ (U/L), median (IQR)	215.5 (154.2-263.5)	177.0 (91.5-221.7)	.05
T-bil^z^ (mg/dL), median (IQR)	0.9 (0.7-1.3)	0.6 (0.4-0.8)	*<.001* ^b^
Glucose (mg/dL), median (IQR)	147.0 (120.5-179.2)	136.0 (113.2-180.5)	.64
CPK^aa^ (U/L), median (IQR)	249.5 (96.5-645.7)	129.0 (80.0-243.0)	.06
AMY^ab^ (U/L), median (IQR)	85.5 (57.7-134.0)	80.5 (61.0-103.5)	.46
CRP^ac^ (mg/dL), median (IQR)	0.4 (0.0-5.5)	0.1 (0.0-1.85)	*.03* ^b^
Daily urinary output (mL), median (IQR)	412.5 (215.0-908.2)	1493.0 (985.0-1977.0)	*<.001* ^b^
Respiratory rate (breaths per minute), median (IQR)	19.5 (17.7-21.0)	16.0 (14.0-20.0)	*.01* ^b^
sBP^ad^ (mmHg), median (IQR)	146.5 (115.5-160.2)	141.0 (124.2-157.0)	.84
dBP^ae^ (mmHg), median (IQR)	81.0 (64.7-88.5)	74.0 (65.5-86.5)	.99
HR^af^ (beats per minute), median (IQR)	97.0 (81.0-113.5)	88.5 (81.2-99.0)	.12
BT^ag^ (°C), median (IQR)	37.0 (36.3-37.5)	37.0 (36.8-37.4)	.23

^a^This table presents the demographic, clinical, and laboratory characteristics of adult patients with burns who were admitted to the ICU at Mie University Hospital between January 2015 and June 2023. This study compared patients who developed ICU delirium with those who did not, highlighting differences in vital signs, burn severity, inflammatory markers, coagulation parameters, organ function indicators, and other physiological variables. The findings aimed to identify key risk factors for ICU delirium and provide insights into the pathophysiology and early prediction of delirium in patients with burns who are critically ill. Statistical comparisons were conducted using median and IQR values, with a *P* value of <.05 considered statistically significant.

^b^Statistically significant at *P*<.05.

^c^WBC: white blood cell count.

^d^RBC: red blood cell count.

^e^MCV: mean corpuscular volume.

^f^MCH: mean corpuscular hemoglobin.

^g^MCHC: mean corpuscular hemoglobin concentration.

^h^APTT: activated partial thromboplastin time.

^i^PT: prothrombin time.

^j^PT percentage.

^k^PT-INR: prothrombin time international normalized ratio.

^l^PCO_2_: partial pressure of carbon dioxide.

^m^SO_2_: saturation of oxygen.

^n^HCO_3_–: bicarbonate.

^o^O_2_-Hb: oxygenated hemoglobin.

^p^CO-Hb: carboxyhemoglobin.

^q^MetHb: methemoglobin.

^r^TP: total protein.

^s^BUN: blood urea nitrogen.

^t^eGFR: estimated glomerular filtration rate.

^u^CL: chloride.

^v^AST: aspartate aminotransferase.

^w^ALT: alanine aminotransferase.

^x^LDH: lactate dehydrogenase.

^y^ALP: alkaline phosphatase.

^z^T-bil: total bilirubin.

^aa^CPK: creatine phosphokinase.

^ab^AMY: amylase.

^ac^CRP: C-reactive protein.

^ad^sBP: systolic blood pressure.

^ae^dBP: diastolic blood pressure.

^af^HR: heart rate.

^ag^BT: body temperature.

Laboratory parameters further demonstrated significant differences. The delirium group showed higher white blood cell counts (median 11.9, IQR 9.0-18.5 per μL vs median 8.8, IQR 6.5-11.3 per μL; *P*=.006), lower hemoglobin (median 13.5, IQR 11.9-15.9 g/dL vs median 14.6, IQR 14.0-15.9 g/dL; *P*=.04), and lower hematocrit levels (median 40%, IQR 35%-44% vs median 43%, IQR 41%-46.7%; *P*=.009). Neutrophil percentages were elevated (median 77%, IQR 73.9%-83.7% vs median 74.4%, IQR 64.6%-80.9%; *P*=.04), whereas lymphocyte (median 15.8%, IQR 8.4%-17.9% vs median 16.8%, IQR 12.6%-23.6%; *P*=.02) and eosinophil (median 0.6%, IQR 0.4%-1.4% vs median 1.2%, IQR 0.6%-1.9%; *P*=.04) percentages were lower in the delirium group. Absolute neutrophil counts were higher (median 8985, IQR 6265-10,830 cells per µL vs median 6840, IQR 4449.2-9029.0 cells per µL; *P*=.03), as were monocyte counts (median 767, IQR 478-993 cells per µL vs median 575.0, IQR 447.5-767.0 cells per µL; *P*=.04).

Coagulation parameters revealed that activated partial thromboplastin time was prolonged in the delirium group (median 30.8, IQR 25.9-36.8 seconds vs median 27.4, IQR 24.9-29.9 seconds; *P*=.02). Prothrombin time (PT) and PT percentage also differed significantly (median 12.2, IQR 11.5-13.6 seconds vs median 11.3, IQR 10.8-12.1 seconds with *P*=.001 and median 94.8%, IQR 77%-104.1% vs median 105.4%, IQR 97%-116.9% with *P*=.001, respectively), as did the PT international normalized ratio (median 1.0, IQR 0.9-1.1 vs median 0.9, IQR 0.9-1.0; *P*=.002). D-dimer levels were elevated (median 4.0, IQR 1.5-8.5 mg/L vs median 1.0, IQR 0.2-8.2 mg/L; *P*=.02).

Acid-base and oxygenation parameters showed a lower pH (median 7.3, IQR 7.3-7.4 vs median 7.4, IQR 7.3-7.4; *P*=.04) and higher PO_2_ (median 152.5, IQR 93.5-322.7 mmHg vs median 123.0, IQR 83.2-166.0 mmHg; *P*=.03) in the delirium group. Oxygen saturation was also significantly higher (median 99.1%, IQR 98.3%-99.6% vs median 97.6%, IQR 94.9%-99%; *P*=.01), as was methemoglobin (MetHb; median 0.9%, IQR 0.5%-1.1% vs median 0.4%, IQR 0.3%-0.9%; *P*=.004).

Biochemical markers indicated lower total protein (median 6.4, IQR 5.9-6.7 g/dL vs median 7.0, IQR 6.2-7.5 g/dL; *P*=.006) and albumin (median 3.6, IQR 3.1-3.9 g/dL vs median 4.1, IQR 3.2-4.4 g/dL; *P*=.004) levels in the delirium group. Creatinine levels were higher (median 0.8, IQR 0.6-1.0 mg/dL vs median 0.7, IQR 0.5-0.8 mg/dL; *P*=.03), and estimated glomerular filtration rate was lower (median 67.2, IQR 47.8-87.2 mL per minute per 1.73 m^2^ vs median 83.7, IQR 59.9-101.0 mL per minute per 1.73 m^2^; *P*=.02). Serum calcium was lower (median 8.5, IQR 8.2-8.9 mg/dL vs median 8.9, IQR 8.4-9.2 mg/dL; *P*=.009), and T-bil levels were elevated (median 0.9, IQR 0.7-1.3 mg/dL vs median 0.6, IQR 0.4-0.8 mg/dL; *P*<.001). C-reactive protein levels were significantly higher in the delirium group (median 0.4, IQR 0.0-5.5 mg/dL vs median 0.1, IQR 0.0-1.85 mg/dL; *P*=.03).

Finally, daily urinary output was markedly reduced in the delirium group (median 412.5, IQR 215.0-908.2 mL vs median 1493.0, IQR 985.0-1977.0 mL; *P*<.001), and respiratory rates were higher (median 19.5, IQR 17.7-21.0 breaths per minute vs median 16.0, IQR 14.0-20.0 breaths per minute; *P*=.01).

### Visual Examination of the Data Using Violin Plots

**Figure 1:**
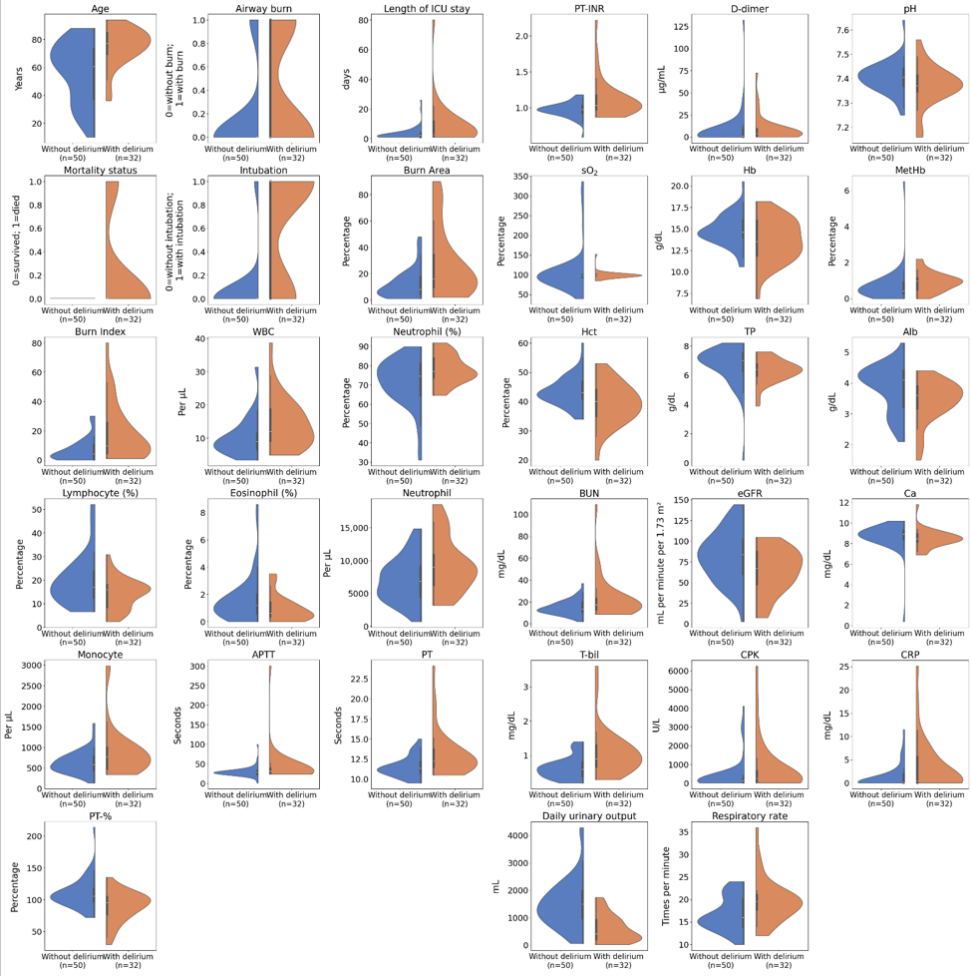
Comparative analysis of explanatory factors for intensive care unit (ICU) delirium in patients with burns using violin plots. This figure illustrates the distribution of key clinical and laboratory variables among 2 groups of ICU patients with burns: those who developed delirium (with delirium) and those who did not (without delirium). Violin plots visualize the distribution and density of each variable, highlighting differences in factors such as age, burn area, total bilirubin (T-bil), methemoglobin (MetHb) levels, daily urinary output, and respiratory rate. These comparisons emphasize the significance of various explanatory factors in predicting ICU delirium, aiding in the identification of potential risk indicators for clinical decision-making. Alb: albumin; APTT: activated partial thromboplastin time; BUN: blood urea nitrogen; Ca: calcium; CPK: creatine phosphokinase; CRP: C-reactive protein; eGFR: estimated glomerular filtration rate; Hb: hemoglobin; Hct: hematocrit level; PT-%: prothrombin time percentage; PT: prothrombin time; PT-INR: PT international normalized ratio; sO2: oxygen saturation; TP: total protein; WBC: white blood cell count.

Next, the distribution of the data between the 2 groups (those with and without ICU delirium) was visually examined using violin plots. As shown in Figure 1, violin plots visually represent the density and range of the data, allowing for the identification of data dispersion and bimodality, that is, the shape of the distribution. Specifically, it became evident that urine output decreased proportionally, serving as a risk factor for ICU delirium.

### Machine Learning Model Evaluation for Delirium Prediction

**Figure 2:**
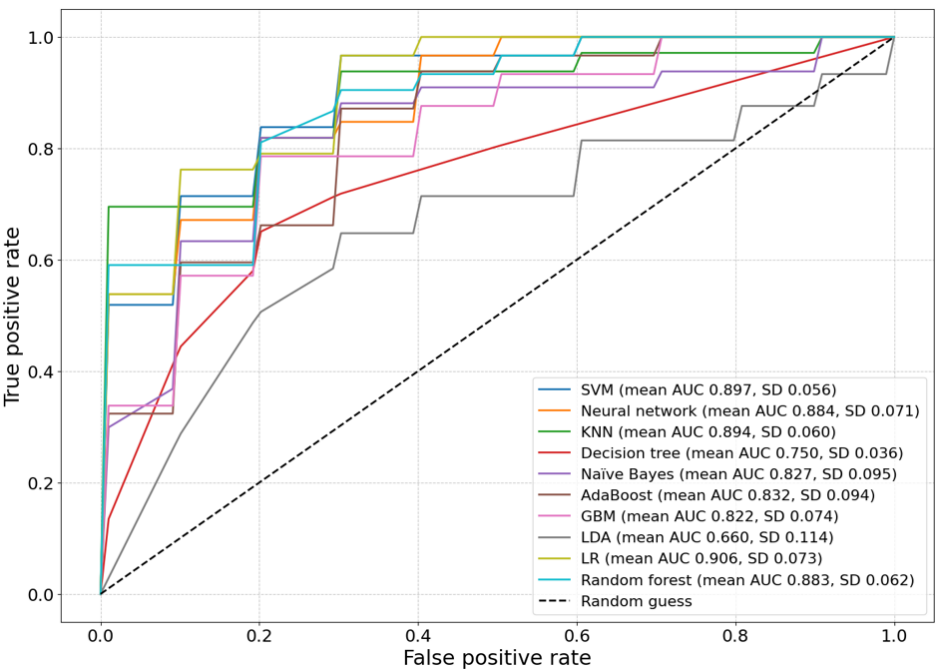
Receiver operating characteristic (ROC) curves for machine learning models predicting intensive care unit (ICU) delirium. This figure shows ROC curves for 10 machine learning models, illustrating sensitivity and 1-specificity from 5-fold stratified cross-validation. The legend presents mean area under the curve (AUC) values with SDs, indicating predictive accuracy. Logistic regression (LR; mean AUC 0.906, SD 0.073) achieved the highest performance, whereas decision tree and linear discriminant analysis (LDA) performed poorly. The diagonal line (AUC 0.5) represents random guessing. Models with curves near the top left corner demonstrate superior predictive ability for ICU delirium in patients with burns. AdaBoost: adaptive boosting; GBM: gradient-boosting machine; KNN: k-nearest neighbor; SVM: support vector machine.

In this study, we evaluated 10 different machine learning models for delirium prediction using 2 performance metrics: the AUC and Matthews correlation coefficient (MCC). The results are summarized in Table 2 and Figure 2.

**Table 2:** Performance comparison of machine learning models across various metrics^a^.

Model	AUC^b^, mean (SD)	MCC^c^, mean (SD)	True positives	True negatives	False positives	False negatives	Accuracy	Precision	Recall	*F*_1_-score
Support vector machine	0.897 (0.056)	0.157 (0.202)	0.6	10.0	0.0	5.8	0.647	0.400	0.095	0.150
Neural network	0.857 (0.058)	0.522 (0.238)	4.2	8.4	1.6	2.2	0.757	0.750	0.652	0.664
K-nearest neighbor	0.894 (0.060)	0.529 (0.311)	3.4	9.6	0.4	3.0	0.793	0.733	0.524	0.605
Decision tree	0.729 (0.033)	0.417 (0.059)	4.2	7.6	2.4	2.2	0.720	0.655	0.624	0.634
Naïve Bayes	0.827 (0.095)	0.411 (0.351)	3.0	9.2	0.8	3.4	0.744	0.683	0.462	0.533
AdaBoost^d^	0.832 (0.094)	0.493 (0.126)	4.0	8.4	1.6	2.4	0.756	0.753	0.614	0.651
Gradient boosting machine	0.821 (0.074)	0.486 (0.026)	3.8	8.6	1.4	2.6	0.768	0.796	0.586	0.651
Linear discriminant analysis	0.660 (0.114)	0.337 (0.163)	3.8	7.4	2.6	2.6	0.684	0.601	0.590	0.583
Logistic regression	*0.906* ^e^ *(0.073)*	*0.625 (0.162)*	4.8	8.6	1.4	1.6	*0.818*	0.797	*0.743*	*0.755*
Random forest	0.850 (0.074)	0.505 (0.217)	3.4	9.2	0.8	3.0	0.757	*0.811*	0.529	0.617

^a^This table presents the predictive performance of 10 machine learning models trained on clinical and laboratory data from patients with burns admitted to the intensive care unit (ICU) at Mie University Hospital. Model performance was evaluated using the mean area under the curve, the mean Matthews correlation coefficient, accuracy, precision, recall, and *F*_1_-score, providing a comprehensive assessment of each model’s ability to predict ICU delirium onset. The table also reports the true positive, true negative, false positive, and false negative counts, offering insights into each model’s sensitivity and specificity. Logistic regression demonstrated the highest predictive performance (area under the curve=0.906), whereas decision tree and linear discriminant analysis showed relatively lower predictive power. These findings highlight the potential of machine learning in early risk stratification for ICU delirium, emphasizing the importance of selecting an optimal predictive model for clinical application.

^b^AUC: area under the curve.

^c^MCC: Matthews correlation coefficient.

^d^AdaBoost: adaptive boosting.

^e^Values in italics indicate the best performance for each metric. Logistic regression exhibited the highest AUC, MCC, accuracy, recall, and *F*₁-score, demonstrating superior overall performance.

The LR model showed the best overall performance, achieving the highest mean AUC of 0.906 (SD 0.073) and the highest mean MCC of 0.625 (SD 0.162). This suggests that LR provides a good balance between discriminative power and classification accuracy for delirium prediction. The SVM and KNN models also showed strong discriminative ability, with mean AUC values of 0.897 (SD 0.056) and 0.894 (SD 0.060), respectively. However, the SVM model’s relatively low MCC score of 0.157 (SD 0.202) indicates that it may struggle with precise classification, possibly due to class imbalance or suboptimal threshold selection. Neural network and RF demonstrated comparable performance, with mean AUC values of 0.857 (SD 0.058) and 0.850 (SD 0.074), respectively. Their MCC scores were also similar, suggesting consistent performance across both metrics. The decision tree model and LDA showed the lowest performance among the evaluated models, with mean AUC values of 0.729 (SD 0.033) and 0.660 (SD 0.114), respectively. This suggests that these models may not capture the complexity of the delirium prediction task as effectively as the other algorithms. It is noteworthy that, while some models (eg, SVM) achieved high AUC scores, their corresponding MCC scores were relatively low. This discrepancy highlights the importance of using multiple evaluation metrics to gain a comprehensive understanding of model performance, especially in potentially imbalanced classification tasks such as delirium prediction. In summary, LR emerged as the most promising model for delirium prediction in this comparative analysis. However, the strong performance of several other models, such as KNN and neural network, suggests that ensemble methods or model stacking could potentially yield further improvements in predictive accuracy (Table 2).

### Performance Metrics of Machine Learning Models in ICU Delirium Prediction

On the basis of the provided classification performance metrics, LR showed the highest overall performance in terms of accuracy (0.818), precision (0.797), recall (0.743), and *F*_1_-score (0.755), indicating a robust balance between sensitivity and specificity. Although KNN showed slightly higher accuracy (0.793) than most models, its recall was lower (0.524), which may limit its utility in detecting positive cases.

Among ensemble methods, GBM and AdaBoost performed well, with GBM achieving a relatively high precision (0.796), reflecting its ability to minimize false positives. RF also yielded strong precision (0.811) but showed lower recall (0.529), suggesting a higher risk of missing positive cases.

Neural network models achieved a competitive balance across all metrics, notably *F*_1_-score (0.664), which highlights their consistency in handling both sensitivity and specificity. However, SVM and LDA demonstrated weaker recall, suggesting that these models are less suited for tasks requiring high sensitivity.

**Figure 3:**
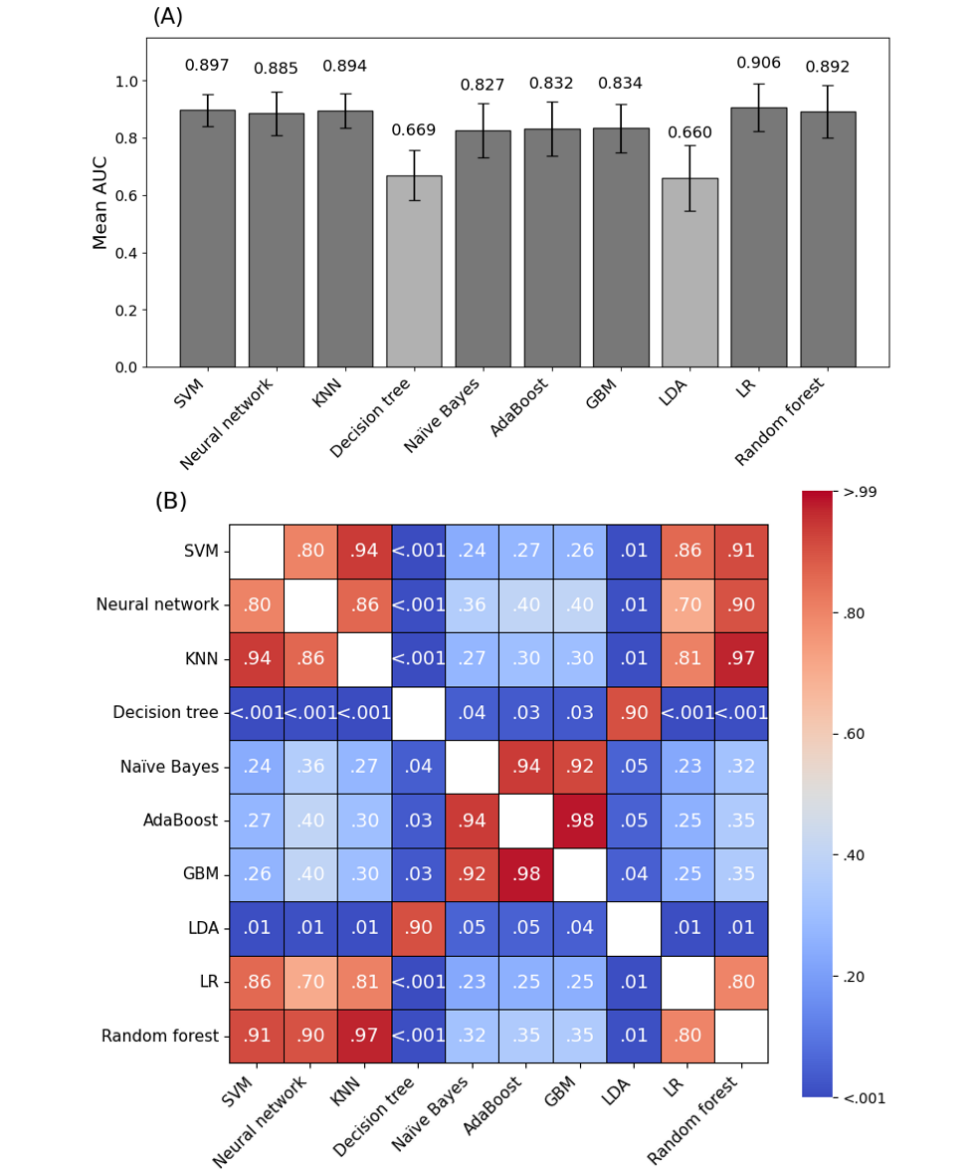
(A) Mean area under the curve (AUC) scores and (B) statistical significance across machine learning models. This figure compares the predictive performance of 10 machine learning models for intensive care unit (ICU) delirium in patients with burns. The bar graph shows mean AUC scores with SDs from 5-fold stratified cross-validation. Among the models, logistic regression (LR; AUC=0.906) performed best, whereas decision tree and linear discriminant analysis (LDA) had lower AUCs. The heat map displays pairwise *P* values from 2-sample 2-tailed t tests, with darker cells indicating significant differences (*P*<.05). This figure highlights model performance and statistical differences, aiding in selecting effective classifiers for ICU delirium prediction. AdaBoost: adaptive boosting; GBM: gradient-boosting machine; KNN: k-nearest neighbor; SVM: support vector machine.

We then compared the mean AUCs across the models to evaluate their overall discriminative ability. When compared with decision tree, SVM (*P*=.002), neural network (*P*=.005), KNN (*P*=.003), LR (*P*=.003), and RF (*P*=.005) showed a significantly better performance. Naïve Bayes (*P*=.04), AdaBoost (*P*=.03), and GBM (*P*=.03) also demonstrated a significantly better performance than that of decision tree. LDA showed a comparable performance to that of decision tree (*P*=.99; Figure 3).

Similarly, when compared with LDA, SVM (*P*=.006), neural network (*P*=.01), KNN (*P*=.007), LR (*P*=.007), and RF (*P*=.009) exhibited a significantly better performance. GBM (*P*=.04) and AdaBoost (*P*=.047) also showed a significantly better performance than that of LDA, whereas naïve Bayes showed a marginally better performance (*P*=.05). Decision tree demonstrated a comparable performance to that of LDA (*P*=.99).

In summary, LR was the most balanced model across all metrics, making it a preferred choice for clinical applications where both precision and recall are critical. Ensemble methods such as GBM and AdaBoost offer strong alternatives, particularly for optimizing precision (Table 2).

### Identification of High-Risk Factors for ICU Delirium Using SHAP Analysis

We used SHAP analysis to identify the top 15 high-risk factors for ICU delirium in each validated machine learning model (Multimedia Appendix 1). This comprehensive analysis revealed distinct sets of risk factors across different models, providing valuable insights into the complex nature of ICU delirium. Key findings included the following:

- LR (best overall performance; AUC=0.906; MCC=0.625): identified daily urine output, eosinophil count, age, and fibrinogen levels as key risk factors.
- Neural network (AUC=0.857; MCC=0.522): highlighted lactate dehydrogenase levels, daily urine output, inhalation injury, neutrophil count, and platelet count as key risk factors.
- SVM (AUC=0.897; MCC=0.157): uniquely identified burn area and length of ICU stay as significant factors.
- KNN (AUC=0.894): emphasized neutrophil and monocyte percentages as key risk factors.
- Decision tree: despite its lower performance, it identified endotracheal intubation and D-dimer levels as risk factors.
- Other models: consistently highlighted hematological parameters, daily urine output, and age across multiple models. Novel factors such as MetHb levels (LDA) and anion gap (RF) were also identified.

## Discussion

### Machine Learning–Based Prediction of ICU Delirium in Patients With Burns: LR Performance and SHAP Analysis for Risk Factor Identification

Our study demonstrated that LR effectively predicted ICU delirium in patients with burns using clinical data, including vital signs and blood biomarkers. Among the machine learning models, LR achieved the highest predictive accuracy, confirming that ICU delirium risk can be assessed through computational modeling (Figures 2 and 3).

Furthermore, SHAP analysis identified key risk factors associated with ICU delirium, highlighting T-bil, MetHb, daily urine output, and leucocyte fractions as novel predictors alongside established factors such as burn area and tracheal intubation (Multimedia Appendix 1). These findings provide crucial insights for developing targeted prevention and treatment strategies, emphasizing the importance of respiratory, renal, hepatic, and inflammatory function management in patients with burns who are critically ill.

### The Role of T-Bil Levels in Predicting Delirium in ICU Patients With Burns: Connections With Cholestasis and Inflammation

T-bil levels were identified as a risk factor for the development of delirium in ICU patients with burns. However, the direct relationship between burns and cholestasis remains unclear. Cholestasis often occurs after burns, and patients with burns who have increased bilirubin levels without a corresponding increase in alkaline phosphatase and gamma-glutamyl transferase levels face a higher risk of mortality. Furthermore, intrahepatic cholestasis is observed in half of patients with severe burns [6]. Cholestasis is also associated with hypoxic hepatitis [7]. In addition, hypovolemic shock observed in severe burns may be involved in the elevation of T-bil levels [8]. Increases in interleukin (IL)-6 and tumor necrosis factor-α, which are observed in the early stages of severe burns [9], have been reportedly associated with hyperbilirubinemia [10] and organ dysfunction [11]. Inflammatory cytokines such as IL-6 and tumor necrosis factor-α may reduce the expression of bile transporters on the canalicular membrane of hepatocytes, leading to an increase in T-bil levels [12,13]. These research findings support the validity of our study, which identified an increase in T-bil levels as an important risk factor for predicting ICU delirium in patients with burns.

### MetHb Level as a Novel Indicator of ICU Delirium in Patients With Burns: Insights From Sepsis and Hemolysis Research

MetHb level was identified as an explanatory factor. No previous studies have clearly demonstrated the involvement of MetHb level as a risk factor for ICU delirium in patients with burns. However, several studies have shown the involvement of MetHb in delirium in patients with sepsis, which, similarly to burns, can cause a cytokine storm. As is well known, patients with sepsis have a high incidence of delirium, and in these patients, nitric oxide is released into the bloodstream due to ischemia-reperfusion stimulation. Nitric oxide is converted into MetHb and nitrates; as a result, the concentration of MetHb in the blood is a useful marker for the onset of sepsis or septic shock [14]. However, the molecular mechanisms through which MetHb causes delirium in patients with sepsis remain largely unknown [15]. In patients with severe conditions, such as trauma or infection, intracellular hemolysis may occur, leading to anemia. Anemia, which develops relatively early in severe conditions, is thought to result from damaged red blood cells processed by the reticuloendothelial system. Acute hemolysis leads to an increase in free hemoglobin in the blood. Subsequently, free hemoglobin and heme are released into the circulatory system, and the wound interstitium is rapidly converted into MetHb by oxidants. The increase in MetHb levels is more pronounced in the ischemia-reperfusion areas, where activated macrophages and neutrophils accumulate [16]. Therefore, MetHb produced by ischemia-reperfusion injury and hemolysis, as observed in severe conditions such as sepsis, may affect leucocyte cell adhesion, phagocytic ability, and metabolic activation and may be involved in ICU delirium. Previous research has shown a stronger correlation among the total amount of hemoglobin [17,18], red blood cell count [19], and delirium. Thus, hemoglobin and MetHb levels in the blood are important factors influencing delirium.

### The Impact of Decreased Urine Output on Delirium Risk in ICU Patients With Burns: Insights From SHAP Analysis

Using SHAP analysis, we identified that a decreased daily urine output within 24 hours of ICU admission is a risk factor for ICU delirium in patients with burns. Interestingly, daily urine output was identified as an important risk factor in 50% (5/10) of the machine learning models evaluated (Multimedia Appendix 1). The finding that a decrease in 24-hour urine volume is a risk factor for delirium in patients with burns seems reasonable considering that acute kidney injury can potentially increase the risk of delirium 10-fold in patients who are critically ill [20]. For example, urine output is an important indicator of renal function and hydration status. In patients with burns, a decrease in urine output may indicate insufficient renal perfusion or dehydration. Both factors contribute to the development of delirium [21]. Furthermore, patients with burns often require multiple medications, such as sedatives and analgesics. Decreased urine output can affect the metabolism and excretion of these drugs, potentially leading to the accumulation of psychoactive substances that can induce or exacerbate delirium [22]. In addition, a decrease in urine output can lead to electrolyte imbalance, which is known to cause neurological dysfunction and delirium. Electrolyte abnormalities such as hyponatremia or hypernatremia can occur in patients with burns because of fluid shifts and inadequate fluid replacement [23].

### Leukocyte Biomarkers as Indicators of Delirium in ICU Patients With Burns: The Role of Inflammatory Response

Our study identified the number or proportion of neutrophils and monocytes in the leucocyte fraction as risk factors for delirium development (Multimedia Appendix 1). This suggests that exposure of leucocytes to a cytokine storm due to excessive stress from burns contributes to delirium in patients with burns. Numerous studies have used leucocyte biomarkers to diagnose delirium in the past [24]. Inflammatory biomarkers and brain-specific metabolic biomarkers have been extensively studied in delirium, and inflammatory cytokines and activation markers of astrocytes and glial cells (IL-6, IL-8, IL-10, tumor necrosis factor-α, C-reactive protein, and S100β protein levels) positively correlate with longer duration of delirium, severity of delirium, and higher in-hospital mortality [25]. In addition, elevated levels of IL-8 and S100β protein have been associated with increased mortality in patients with delirium [26]. In a mouse model with delirium, the infiltration of bone marrow–derived monocytes into the blood-brain barrier [27] and activation of microglia were observed [28]. Although the number and proportion of neutrophils and monocytes were identified as risk factors for ICU delirium, these leucocytes may be involved in the production of inflammatory cytokines and contribute to the onset of ICU delirium.

### Respiratory Rate as a Predictor of ICU Delirium in Patients With Burns: New Insights and Implications

In our study, an increase in the respiratory rate was a risk factor for ICU delirium. Patients with ICU delirium had a higher median respiratory rate of 19.5 (IQR 17.7-21.0) breaths per minute compared to 16.0 (IQR 14.0-20.0) breaths per minute in patients without delirium (*P*=.01; Table 1). To our knowledge, no studies have clearly established a link between delirium and respiratory rate. Delirium is generally recognized as a common complication in patients with respiratory failure in the ICU. The incidence of delirium in the ICU ranges from 10% to 78%, with most cases occurring in patients receiving mechanical ventilation. This suggests a significant overlap between respiratory complications and the occurrence of delirium; however, a direct correlation between an increase in respiratory rate and delirium has not been explicitly stated [29]. Inhalation injuries occur in approximately one-third of burn hospital admissions and contribute to a high mortality rate (50%) in patients with burns. Therefore, an increase in the respiratory rate may be associated with carbon monoxide poisoning and chemical tracheobronchitis due to the inhalation of toxic combustion products and generally correlates with a higher mortality rate. Unfortunately, many patients with burns receive high-concentration oxygen therapy from emergency teams before being transported to the ICU or emergency room. Therefore, carboxyhemoglobin levels are often adjusted to lower levels, and PO_2_ is frequently high during treatment, which is why carboxyhemoglobin and PO_2_ were not identified as risk factors in our model. Therefore, it might be appropriate to consider respiratory rate as a potential risk factor for ICU delirium in future studies [30,31].

### Advancing ICU Delirium Research: The Prediction of Delirium in ICU Patients Model and the Need for Machine Learning Approaches in Patients With Burns

In the field of delirium research, the Prediction of Delirium in ICU Patients (PRE-DELIRIC) model is considered a seminal study [32]. In the ICU, the PRE-DELIRIC model uses 10 identified risk factors (age, Acute Physiology and Chronic Health Evaluation II score, admission group, coma, infection, metabolic acidosis, use of sedatives and morphine, blood urea nitrogen, and emergency admission) and predicts delirium with an AUC of 0.87 (95% CI 0.85-0.89) within 24 hours of ICU admission [32]. Furthermore, the model by Lanzhou University [33] heavily relies on patients’ detailed past medical histories, making data collection challenging in busy clinical settings such as emergency rooms and ICUs, where obtaining comprehensive patient histories, diagnoses, and treatments can be difficult [34]. Therefore, it is crucial to establish machine learning models that can accurately predict conditions with multifactorial risk factors, such as ICU delirium, using data that are easily obtainable during emergency department visits, such as vital signs and blood data. Despite this need, a delirium prediction model for ICU patients with burns using machine learning has not yet been developed. Predictive models for diseases such as ICU delirium, which involve numerous risk factors, stand to benefit significantly from machine learning’s capability to perform multifactorial analyses, surpassing traditional biostatistical methods. Our proposed machine learning model can more effectively evaluate complex interactions among multivariate data, which is essential for accurately predicting conditions with multifactorial risk factors such as ICU delirium. Therefore, although our study had a small number of cases, it is considered valuable for the development of a machine learning–based ICU delirium prediction model and the identification of risk factors.

### Comprehensive Model Evaluation for ICU Delirium

When evaluating the performance of different models, it is important to consider not only the AUC but also other metrics, such as accuracy, precision, recall, and *F*_1_-score. In a specialized medical environment such as the ICU, some metrics may become more important than others when dealing with specific diseases. For example, precision is important if avoiding false detections of delirium is crucial. Conversely, if it is vital to avoid missing cases of delirium, recall should be emphasized. In our study, we considered all these metrics comprehensively and selected the model that best suited the objectives of the research and clinical demands.

Among the 10 models we adjusted, the LR model was found to be the most balanced and high performing. Notably, it demonstrated the highest values across key metrics, including AUC, MCC, accuracy, and *F*_1_-score, suggesting that it provides the most reliable predictions from various perspectives.

### Strengths

The explanatory variables in our study were based on blood data collected immediately upon the arrival of patients with burns at the emergency outpatient clinic. Therefore, there was a time lag between the collection of these data and the collection of data at the time of delirium diagnosis using the ICDSC. However, we believe that our model, which accurately predicts the development of delirium during an ICU stay based on blood data and vital signs at the time of patient arrival, can be easily interpreted by clinicians and has high general applicability. This allows clinicians to predict the incidence of ICU delirium, which significantly affects the prognosis of severe burns from early in the patient’s hospitalization, enabling the initiation of early interventions for patients at high risk of ICU delirium.

### Limitations

#### Demerit of Binary Classification for Delirium

A binary classification model was used for predicting delirium, which simplified the model’s output to either *delirium present* or *delirium absent*. This approach facilitated decision-making in clinical settings by providing clear, binary outcomes. Conversely, using a regression model would require predicting specific ICDSC scores and interpreting these scores to assess patient status. Thus, binary classification often offers greater practicality in busy clinical environments due to its straightforward interpretation. However, regression analysis may be more appropriate for predicting continuous outcomes, such as the ICDSC score. This method offers detailed information by producing continuous values, allowing for a nuanced understanding of the severity of ICU delirium, ranging from mild to severe. For instance, the difference in delirium between an ICDSC score of 3 and 4 might be minimal, whereas the difference between scores of 1 and 7 indicates substantially different symptoms. Therefore, future research should investigate the benefits of developing regression models that predict ICDSC scores.

#### Model Selection Criteria for Small Datasets

This study examined delirium in patients with burns who were critically ill admitted to the ICU and did not include patients with missing data, resulting in a small number of cases. In analyses with small datasets, such as in our 82 cases, it is generally recommended to avoid overfitting and choose a simpler model. In this study, we were cautious about using complex models (such as deep neural networks or RF with many trees) that are prone to overfitting with limited data. Therefore, simple models are generally recommended for studies with few cases. Typically, models with regularization effects (such as LR) are effective in preventing overfitting. In our study, LR demonstrated a high accuracy (0.818), indicating that overfitting was well controlled during model creation. Linear SVMs have also been proposed to prevent overfitting. In addition, LDA has linear boundaries, is computationally fast, and can sometimes provide relatively stable results even with a small amount of data.

#### Limitations and Future Directions for External Validation

The performance of our model was discussed using data from patients in the ICU in a single hospital; however, an external validation was not conducted. Therefore, our study is limited by its single-institution setting. Future research could strengthen the reliability of our delirium prediction model for patients with burns in the ICU by conducting external validation using datasets from other hospitals.

#### Enhancing Predictive Power: Inclusion of Diverse Variables

Our machine learning model incorporated 70 explanatory variables, including patients’ vital signs and blood biomarkers. However, unlike the PRE-DELIRIC model, which includes medication history and environmental factors, our study did not consider these variables. Expanding the range of input variables in future research may further enhance the predictive accuracy of delirium risk assessment.

### Conclusions

Our study underscores the clinical utility of machine learning in predicting ICU delirium in patients with burns, demonstrating that LR provided the highest predictive accuracy among the tested models. Using SHAP analysis, we identified both well-established and novel risk factors, such as T-bil, MetHb, urine output, and leucocyte fractions, offering new insights into the complex pathophysiology of delirium. These findings suggest that early identification of patients at high risk using readily available clinical data upon ICU admission could facilitate proactive intervention strategies, potentially reducing morbidity and improving patient outcomes.

Beyond its immediate clinical applications, this study highlights the need for integrating machine learning into real-world ICU decision-making systems. Traditional delirium prediction models such as PRE-DELIRIC rely on a limited set of predefined variables, whereas machine learning models can dynamically incorporate diverse clinical parameters, enabling real-time risk stratification. This adaptability is particularly relevant in burn care, where patients exhibit highly variable and rapidly evolving physiological changes.

Moving forward, the integration of multi-institutional external validation is crucial to ensure the generalizability and robustness of our predictive model across diverse ICU settings. In addition, further research should explore the mechanistic pathways linking identified risk factors with delirium onset, which could pave the way for personalized prevention and treatment strategies. Ultimately, this study provides a foundation for the next generation of delirium risk prediction models, emphasizing the potential of artificial intelligence–driven clinical decision support to enhance patient care in critical care medicine.

## Appendix

Multimedia Appendix 1 Comparison of feature Shapley additive explanations (SHAP) values across multiple machine learning models. The 15 most influential features in each model’s predictions are displayed. A higher SHA*P* value indicates a stronger impact of the feature on the model’s predictions.

Multimedia Appendix 2 Python code used in this study for generating the violin plots comparing explanatory factors for intensive care unit delirium in patients with burns.

Multimedia Appendix 3 Python code used in this study for generating the receiver operating characteristic curves illustrating machine learning model performance in predicting intensive care unit delirium.

Multimedia Appendix 4 Python code used in this study for generating the bar graph and heat map comparing machine learning model performance in predicting intensive care unit delirium.

Multimedia Appendix 5 Adaptive boosting code for Shapley additive explanations analysis.

Multimedia Appendix 6 Decision tree code for Shapley additive explanations analysis.

Multimedia Appendix 7 K-nearest neighbor code for Shapley additive explanations analysis.

Multimedia Appendix 8 Linear discriminant analysis code for Shapley additive explanations analysis.

Multimedia Appendix 9 Light gradient-boosting machine code for Shapley additive explanations analysis.

Multimedia Appendix 10 Logistic regression code for Shapley additive explanations analysis.

Multimedia Appendix 11 Naïve Bayes code for Shapley additive explanations analysis.

Multimedia Appendix 12 Neural network code for Shapley additive explanations analysis.

Multimedia Appendix 13 Random forest code for Shapley additive explanations analysis.

Multimedia Appendix 14 Support vector machine code for Shapley additive explanations analysis.

## Abbreviations

AdaBoost: adaptive boosting
AUC: area under the receiver operating characteristic curve
CAM-ICU: Confusion Assessment Method for the Intensive Care Unit
GBM: gradient-boosting machine
ICDSC: Intensive Care Delirium Screening Checklist
ICU: intensive care unit
IL: interleukin
LDA: linear discriminant analysis
LR: logistic regression
MCC: Matthews correlation coefficient
MetHb: methemoglobin
PRE-DELIRIC: Prediction of Delirium in Intensive Care Unit Patients
PT: prothrombin time
RF: random forest
SHAP: Shapley additive explanations
SVM: support vector machine
T-bil: total bilirubin

## Data Availability

The datasets generated or analyzed during this study are not publicly available due to the need to protect participant confidentiality but are available from the corresponding author on reasonable request. In addition, the Python code used for the machine learning analyses, including modifications and optimizations conducted using generative artificial intelligence, is provided in Multimedia Appendices 2-14. Further details regarding the code can be obtained by contacting the corresponding author.
