# Supplementary material for: Machine Learning–Based Prediction of Delirium and Risk Factor Identification in Intensive Care Unit Patients With Burns: Retrospective Observational Study
Source: JMIR Form Res. 2025 Mar 5;9:e65190. doi: 10.2196/65190 (PMC11923481; doi:10.2196/65190)
Supplement: Multimedia Appendix 1 [file formative_v9i1e65190_app1.docx]

| Feature | | SHAP value |
| --- | --- | --- |
| **Logistic regression** | | |
|  | Daily urinary output | 8.9507 |
|  | Eosinophil count | 7.384 |
|  | Age | 4.701 |
|  | Basophil count | 4.3624 |
|  | Fibrinogen | 3.5751 |
|  | dBP^a^ | 3.0054 |
|  | PO_2_ | 2.5398 |
|  | CPK^b^ | 2.2675 |
|  | LDH^c^ | 2.2588 |
|  | AST^d^ | 2.2078 |
|  | sBP^e^ | 1.9375 |
|  | Glucose | 1.7221 |
|  | Height | 1.6423 |
|  | ALP^f^ | 1.5095 |
|  | Burn area | 1.4838 |
| **SVM^g^** | | |
|  | Age | 0.0423 |
|  | Height | 0.0388 |
|  | PCO_2_^h^ | 0.0292 |
|  | Burn area | 0.0214 |
|  | Length of ICU^i^ stay | 0.0214 |
|  | Basophil count | 0.0211 |
|  | dBP | 0.0197 |
|  | Burn index | 0.0155 |
|  | BUN^j^ | 0.0151 |
|  | CRP^k^ | 0.0109 |
|  | HCO_3_–^l^ | 0.0103 |
|  | Sodium | 0.0098 |
|  | ALT^m^ | 0.009 |
|  | Fibrinogen | 0.0086 |
|  | CO-Hb^n^ | 0.0084 |
| **Neural network** | | |
|  | Neutrophil count | 0.0212 |
|  | LDH | 0.0186 |
|  | Daily urinary output | 0.0123 |
|  | Airway burn | 0.0102 |
|  | Platelet count | 0.0099 |
|  | Burn index | 0.0097 |
|  | Height | 0.0095 |
|  | Mortality status | 0.0086 |
|  | Creatinine | 0.0086 |
|  | MCV^o^ | 0.0084 |
|  | TP^p^ | 0.0081 |
|  | AMY^q^ | 0.0077 |
|  | PCO_2_ | 0.0075 |
|  | Lymphocytes (%) | 0.0074 |
|  | WBC^r^ | 0.006 |
| **LDA^s^** | | |
|  | pH | 291.6668 |
|  | RBC^t^ | 57.5817 |
|  | Calcium | 30.515 |
|  | Eosinophils (%) | 26.5442 |
|  | MetHb^u^ | 24.045 |
|  | Intubation | 21.8692 |
|  | PT^v^ | 19.8544 |
|  | Creatinine | 16.428 |
|  | MCHC^w^ | 8.4963 |
|  | Airway burn | 8.0179 |
|  | Gender | 7.3941 |
|  | CO-Hb | 4.9932 |
|  | PCO_2_ | 4.9787 |
|  | Anion gap | 4.259 |
|  | CRP | 3.8403 |
| **Decision tree** | | |
|  | Intubation | 0.4411 |
|  | D-dimer | 0.1684 |
|  | Age | 0.1514 |
|  | Monocytes (%) | 0.0991 |
|  | RBC | 0.0758 |
|  | Chloride | 0.0642 |
|  | CO-Hb | 0 |
|  | O_2_-Hb^x^ | 0 |
|  | MetHb | 0 |
|  | Ionized calcium | 0 |
|  | Hemoglobin | 0 |
|  | Hematocrits | 0 |
|  | TP | 0 |
|  | Albumin | 0 |
|  | BUN | 0 |
| **Naïve Bayes** | | |
|  | Neutrophil count | 2351.3516 |
|  | Daily urinary output | 1000.7388 |
|  | CPK | 579.8882 |
|  | LDH | 318.6199 |
|  | Monocyte count | 270.4776 |
|  | Lymphocyte count | 161.122 |
|  | AST | 70.9116 |
|  | AMY | 68.248 |
|  | PO_2_ | 63.3181 |
|  | Eosinophil count | 58.6545 |
|  | Platelet count | 44.9096 |
|  | ALP | 28.4197 |
|  | eGFR^y^ | 24.6422 |
|  | Age | 19.4695 |
|  | ALT | 18.0366 |
| **AdaBoost^z^** | | |
|  | Daily urinary output | 0.1344 |
|  | Age | 0.1239 |
|  | SO_2_^aa^ | 0.0712 |
|  | Intubation | 0.0643 |
|  | Hematocrits | 0.0575 |
|  | PCO_2_ | 0.0531 |
|  | MCHC | 0.0498 |
|  | T-bil^ab^ | 0.0419 |
|  | TP | 0.0376 |
|  | O_2_-Hb | 0.0323 |
|  | Burn index | 0.0317 |
|  | Length of ICU stay | 0.0305 |
|  | dBP | 0.0304 |
|  | Fibrinogen | 0.0297 |
|  | Eosinophil count | 0.0252 |
| **KNN^ac^** | | |
|  | PO_2_ | 0.1353 |
|  | PCO_2_ | 0.1 |
|  | Neutrophils (%) | 0.0941 |
|  | ALP | 0.0706 |
|  | Monocytes (%) | 0.0706 |
|  | Albumin | 0.0706 |
|  | BMI | 0.0647 |
|  | T-bil | 0.0647 |
|  | Platelet count | 0.0647 |
|  | Daily urinary output | 0.0588 |
|  | MCH^ad^ | 0.0588 |
|  | eGFR | 0.0588 |
|  | Lymphocytes (%) | 0.0529 |
|  | WBC | 0.0529 |
|  | MCV | 0.0529 |
| **Random forest** | | |
|  | Intubation | 0.0873 |
|  | Daily urinary output | 0.0576 |
|  | Hematocrits | 0.0505 |
|  | Anion gap | 0.0291 |
|  | Hemoglobin | 0.0284 |
|  | PCO_2_ | 0.0258 |
|  | WBC | 0.0247 |
|  | Age | 0.024 |
|  | APTT^ae^ | 0.021 |
|  | Length of ICU stay | 0.0204 |
|  | PT | 0.0201 |
|  | HCO_3_– | 0.0199 |
|  | PO_2_ | 0.0197 |
|  | SO_2_ | 0.0192 |
|  | Monocyte count | 0.0191 |
| **LightGBM^af^** | | |
|  | Daily urinary output | 28 |
|  | Age | 22 |
|  | T-bil | 18 |
|  | Intubation | 18 |
|  | SO_2_ | 18 |
|  | PCO_2_ | 10 |
|  | Ionized calcium | 7 |
|  | BMI | 6 |
|  | D-dimer | 5 |
|  | pH | 5 |
|  | Albumin | 5 |
|  | Hematocrits | 5 |
|  | Monocytes (%) | 4 |
|  | CO-Hb | 4 |
|  | TP | 3 |

^a^dBP: diastolic blood pressure.

^b^CPK: creatine phosphokinase.

^c^LDH: lactate dehydrogenase.

^d^AST: aspartate aminotransferase.

^e^sBP: systolic blood pressure.

^f^ALP: alkaline phosphatase.

^g^SVM: support vector machine.

^h^PCO_2_: partial pressure of carbon dioxide.

^i^ICU: intensive care unit.

^j^BUN: blood urea nitrogen.

^k^CRP: C-reactive protein.

^l^HCO_3_–: bicarbonate.

^m^ALT: alanine aminotransferase.

^n^CO-HB: carboxyhemoglobin.

^o^MCV: mean corpuscular volume.

^p^TP: total protein.

^q^AMY: amylase.

^r^WBC: white blood cell count.

^s^LDA: linear discriminant analysis.

^t^RBC: red blood cell count.

^u^MetHb: methemoglobin.

^v^PT: prothrombin time.

^w^MCHC: mean corpuscular hemoglobin concentration.

^x^O_2_-Hb: oxygenated hemoglobin.

^y^eGFR: estimated glomerular filtration rate.

^z^AdaBoost: Adaptive Boosting.

^aa^SO_2_: saturation of oxygen.

^ab^T-bil: total bilirubin.

^ac^KNN: k-nearest neighbor.

^ad^MCH: mean corpuscular hemoglobin.

^ae^APTT: activated partial thromboplastin time.

^af^LightGBM: Light Gradient-Boosting Machine.
