# Supplementary material for: Machine Learning–Based Prediction of Delirium and Risk Factor Identification in Intensive Care Unit Patients With Burns: Retrospective Observational Study
Source: JMIR Form Res. 2025 Mar 5;9:e65190. doi: 10.2196/65190 (PMC11923481; doi:10.2196/65190)
Supplement: Multimedia Appendix 2 [file formative_v9i1e65190_app2.docx]

import pandas as pd

import seaborn as sns

import matplotlib.pyplot as plt

# Load the dataset (specify the correct file path)

data = pd.read_csv("/content/drive", encoding="shift_jis")

# List of explanatory variables and their units

variables = [

    ('Age', 'years'), ('Airway burn', '0:without burn, 1:with burn'), ('ICU length of stay', 'days'),

    ('Dead', '0:alive, 1:dead'), ('Intubation', '0:without intubation, 1:with intubation'),

    ('Burn Area', '%'), ('Burn Index', ''), ('WBC', '/μL'), ('Neutrophil(%)', '%'),

    ('Lymphocyte(%)', '%'), ('Eosinopihl(%)', '%'), ('Neutrophil', '/μL'), ('Monocyte', '/μL'),

    ('APTT', 'seconds'), ('PT', 'seconds'), ('PT-%', '%'), ('PT-INR', ''), ('D-dimer', 'μg/mL'),

    ('pH', ''), ('sO2', '%'), ('Hb', 'g/dL'), ('Met-Hb', '%'), ('Hct', '%'), ('TP', 'g/dL'),

    ('Alb', 'g/dL'), ('BUN', 'mg/dL'), ('eGFR', 'mL/min/1.73m²'), ('Ca', 'mg/dL'),

    ('T-Bil', 'mg/dL'), ('CPK', 'U/L'), ('CRP', 'mg/dL'), ('Daily urinary output', 'mL'),

    ('Respiratory rate', 'times/min')

]

# Count the number of data points with and without delirium

count_without_delirium = data[data["Delirium"] == 0].shape[0]

count_with_delirium = data[data["Delirium"] == 1].shape[0]

# Split the list of explanatory variables into two parts

mid_idx = len(variables) // 2

variables1 = variables[:mid_idx]

variables2 = variables[mid_idx:]

def plot_violins(variables_list, fig_num):

    # Set up the layout for displaying the plots

    n_rows = (len(variables_list) + 2) // 3

    fig, axes = plt.subplots(n_rows, 3, figsize=(18, n_rows * 6))

    # Plot violin plots for each explanatory variable

    for i, (var, unit) in enumerate(variables_list):

        row = i // 3

        col = i % 3

        ax = axes[row, col]

        # Add the cut parameter to ignore the effect of KDE

        sns.violinplot(x="Delirium", y=var, data=data, palette="muted", split=True, ax=ax, cut=0)

        # Change the x-axis labels (split into two lines)

        ax.set_xticklabels([f'Without Delirium\n(n={count_without_delirium})', f'With Delirium\n(n={count_with_delirium})'])

        ax.set_xlabel("")  # Remove x-axis label

        # Add y-axis label

        ax.set_ylabel(unit, fontsize=22.5)

        # Adjust title font size (double size)

        ax.set_title(var, fontsize=22.5)  # Double the title font size

        # Adjust tick label font size

        ax.tick_params(labelsize=24)  # Double the y-axis tick label font size

        # Set the font size for x-axis labels

        for label in ax.get_xticklabels():

            label.set_fontsize(18)

    # Hide any extra subplots

    for i in range(len(variables_list), n_rows * 3):

        row = i // 3

        col = i % 3

        axes[row, col].axis('off')

    plt.tight_layout()

    plt.show()

# Plot the two figures

plot_violins(variables1, 1)

plot_violins(variables2, 2)
