# Supplementary material for: Machine Learning–Based Prediction of Delirium and Risk Factor Identification in Intensive Care Unit Patients With Burns: Retrospective Observational Study
Source: JMIR Form Res. 2025 Mar 5;9:e65190. doi: 10.2196/65190 (PMC11923481; doi:10.2196/65190)
Supplement: Multimedia Appendix 3 [file formative_v9i1e65190_app3.docx]

import pandas as pd

import numpy as np

from sklearn.model_selection import StratifiedKFold, GridSearchCV

from sklearn.preprocessing import StandardScaler

from sklearn.impute import SimpleImputer

from sklearn.metrics import roc_auc_score, roc_curve, matthews_corrcoef

import matplotlib.pyplot as plt

# Data loading and preprocessing

data = pd.read_csv("/content/drive", encoding="shift_jis")

imputer = SimpleImputer(strategy='mean')

data_imputed = pd.DataFrame(imputer.fit_transform(data), columns=data.columns)

X = data_imputed.drop(columns=["Delirium"])

y = data_imputed["Delirium"]

# Model imports

from sklearn.svm import SVC

from sklearn.neural_network import MLPClassifier

from sklearn.neighbors import KNeighborsClassifier

from sklearn.tree import DecisionTreeClassifier

from sklearn.naive_bayes import GaussianNB

from sklearn.ensemble import AdaBoostClassifier, GradientBoostingClassifier, RandomForestClassifier

from sklearn.discriminant_analysis import LinearDiscriminantAnalysis

from sklearn.linear_model import LogisticRegression

# Define hyperparameter grids

param_grids = {

"SVM": {

'C': [0.1, 1, 10, 100],

'kernel': ['rbf', 'linear', 'poly', 'sigmoid'],

'gamma': ['scale', 'auto', 0.1, 1],

'degree': [2, 3, 4],

'coef0': [0.0, 0.1, 0.5]

},

"Neural Network": {'hidden_layer_sizes': [(50,), (100,), (50, 50)], 'alpha': [0.0001, 0.001, 0.01]},

"k-NN": {'n_neighbors': [3, 5, 7], 'weights': ['uniform', 'distance']},

"Decision Tree": {'max_depth': [None, 5, 10], 'min_samples_split': [2, 5, 10]},

"Random Forest": {'n_estimators': [50, 100, 200], 'max_depth': [None, 5, 10]}

}

# Initialize models

models = {

"SVM": SVC(probability=True),

"Neural Network": MLPClassifier(max_iter=1000),

"k-NN": KNeighborsClassifier(),

"Decision Tree": DecisionTreeClassifier(),

"Naive Bayes": GaussianNB(),

"AdaBoost": AdaBoostClassifier(),

"GBM": GradientBoostingClassifier(),

"LDA": LinearDiscriminantAnalysis(),

"LR": LogisticRegression(),

"Random Forest": RandomForestClassifier()

}

# Set up stratified K-fold cross-validation

n_splits = 5

skf = StratifiedKFold(n_splits=n_splits, shuffle=True, random_state=42)

# Dictionary to store results

results = {name: {'auc': [], 'mcc': []} for name in models.keys()}

best_params = {}

roc_curves = {}

# Standardize the data

scaler = StandardScaler()

X_scaled = scaler.fit_transform(X)

# Perform grid search and cross-validation

for name, model in models.items():

if name in param_grids:

grid_search = GridSearchCV(model, param_grids[name], cv=skf, scoring='roc_auc', n_jobs=-1)

grid_search.fit(X_scaled, y)

best_model = grid_search.best_estimator_

best_params[name] = grid_search.best_params_

else:

best_model = model

# Calculate AUC and MCC in cross-validation

mean_fpr = np.linspace(0, 1, 100)

tprs = []

aucs = []

mccs = []

for train_index, test_index in skf.split(X_scaled, y):

X_train, X_test = X_scaled[train_index], X_scaled[test_index]

y_train, y_test = y.iloc[train_index], y.iloc[test_index]

best_model.fit(X_train, y_train)

y_pred_prob = best_model.predict_proba(X_test)[:, 1]

y_pred = best_model.predict(X_test)

auc = roc_auc_score(y_test, y_pred_prob)

mcc = matthews_corrcoef(y_test, y_pred)

aucs.append(auc)

mccs.append(mcc)

# Create ROC curve

fpr, tpr, _ = roc_curve(y_test, y_pred_prob)

tprs.append(np.interp(mean_fpr, fpr, tpr))

tprs[-1][0] = 0.0

mean_tpr = np.mean(tprs, axis=0)

mean_tpr[-1] = 1.0

mean_auc = np.mean(aucs)

std_auc = np.std(aucs)

mean_mcc = np.mean(mccs)

std_mcc = np.std(mccs)

results[name]['auc'] = aucs

results[name]['mcc'] = mccs

roc_curves[name] = (mean_fpr, mean_tpr, mean_auc, std_auc)

# Visualize ROC curves

plt.figure(figsize=(12, 8))

for name, (mean_fpr, mean_tpr, mean_auc, std_auc) in roc_curves.items():

plt.plot(mean_fpr, mean_tpr, label=f'{name} (AUC = {mean_auc:.3f} ± {std_auc:.3f})')

plt.plot([0, 1], [0, 1], 'k--', label='Random Guess')

plt.xlabel('False Positive Rate')

plt.ylabel('True Positive Rate')

plt.title('Mean ROC Curves with AUC')

plt.legend(loc='lower right')

plt.tight_layout()

plt.show()

# Display results

for name, metrics in results.items():

mean_auc = np.mean(metrics['auc'])

std_auc = np.std(metrics['auc'])

mean_mcc = np.mean(metrics['mcc'])

std_mcc = np.std(metrics['mcc'])

print(f"{name}:")

print(f" Mean AUC = {mean_auc:.3f} (±{std_auc:.3f})")

print(f" Mean MCC = {mean_mcc:.3f} (±{std_mcc:.3f})")

if name in best_params:

print(f" Best parameters: {best_params[name]}")

print()
