# Supplementary material for: Machine Learning–Based Prediction of Delirium and Risk Factor Identification in Intensive Care Unit Patients With Burns: Retrospective Observational Study
Source: JMIR Form Res. 2025 Mar 5;9:e65190. doi: 10.2196/65190 (PMC11923481; doi:10.2196/65190)
Supplement: Multimedia Appendix 4 [file formative_v9i1e65190_app4.docx]

import pandas as pd

import numpy as np

from sklearn.model_selection import StratifiedKFold, GridSearchCV

from sklearn.preprocessing import StandardScaler

from sklearn.impute import SimpleImputer

from sklearn.metrics import roc_auc_score

from scipy.stats import ttest_ind, f_oneway

import matplotlib.pyplot as plt

# Data loading and preprocessing

data = pd.read_csv("/content/drive", encoding="shift_jis")

imputer = SimpleImputer(strategy='mean')

data_imputed = pd.DataFrame(imputer.fit_transform(data), columns=data.columns)

X = data_imputed.drop(columns=["Delirium"])

y = data_imputed["Delirium"]

# Model imports

from sklearn.svm import SVC

from sklearn.neural_network import MLPClassifier

from sklearn.neighbors import KNeighborsClassifier

from sklearn.tree import DecisionTreeClassifier

from sklearn.naive_bayes import GaussianNB

from sklearn.ensemble import AdaBoostClassifier, GradientBoostingClassifier, RandomForestClassifier

from sklearn.discriminant_analysis import LinearDiscriminantAnalysis

from sklearn.linear_model import LogisticRegression

# Define hyperparameter grids

param_grids = {

"SVM": {

'C': [0.1, 1, 10, 100],

'kernel': ['rbf', 'linear', 'poly', 'sigmoid'],

'gamma': ['scale', 'auto', 0.1, 1],

'degree': [2, 3, 4], # Degree of the polynomial kernel

'coef0': [0.0, 0.1, 0.5] # Parameter for poly and sigmoid kernels

},

"Neural Network": {'hidden_layer_sizes': [(50,), (100,), (50, 50)], 'alpha': [0.0001, 0.001, 0.01]},

"k-NN": {'n_neighbors': [3, 5, 7], 'weights': ['uniform', 'distance']},

"Decision Tree": {'max_depth': [None, 5, 10], 'min_samples_split': [2, 5, 10]},

"Random Forest": {'n_estimators': [50, 100, 200], 'max_depth': [None, 5, 10]}

}

# Initialize models

models = {

"SVM": SVC(probability=True),

"Neural Network": MLPClassifier(max_iter=1000),

"k-NN": KNeighborsClassifier(),

"Decision Tree": DecisionTreeClassifier(),

"Naive Bayes": GaussianNB(),

"AdaBoost": AdaBoostClassifier(),

"GBM": GradientBoostingClassifier(),

"LDA": LinearDiscriminantAnalysis(),

"LR": LogisticRegression(),

"Random Forest": RandomForestClassifier()

}

# Stratified K-Fold cross-validation setup

n_splits = 5

skf = StratifiedKFold(n_splits=n_splits, shuffle=True, random_state=42)

# Dictionary to store results

results = {name: [] for name in models.keys()}

best_params = {}

# Standardize the data

scaler = StandardScaler()

X_scaled = scaler.fit_transform(X)

# Grid search and cross-validation execution

for name, model in models.items():

if name in param_grids:

grid_search = GridSearchCV(model, param_grids[name], cv=skf, scoring='roc_auc', n_jobs=-1)

grid_search.fit(X_scaled, y)

best_model = grid_search.best_estimator_

best_params[name] = grid_search.best_params_

else:

best_model = model

# Calculate AUC during cross-validation

for train_index, test_index in skf.split(X_scaled, y):

X_train, X_test = X_scaled[train_index], X_scaled[test_index]

y_train, y_test = y.iloc[train_index], y.iloc[test_index]

best_model.fit(X_train, y_train)

y_pred_prob = best_model.predict_proba(X_test)[:, 1]

auc = roc_auc_score(y_test, y_pred_prob)

results[name].append(auc)

# Display results

for name, aucs in results.items():

mean_auc = np.mean(aucs)

std_auc = np.std(aucs)

print(f"{name}: Mean AUC = {mean_auc:.3f} (±{std_auc:.3f})")

if name in best_params:

print(f" Best parameters: {best_params[name]}")

print()

# Visualization

plt.figure(figsize=(12, 7))

for name, aucs in results.items():

mean_auc = np.mean(aucs)

std_auc = np.std(aucs)

plt.bar(name, mean_auc, yerr=std_auc, capsize=5, alpha=0.7)

plt.text(name, mean_auc + std_auc + 0.02, f'{mean_auc:.3f}', ha='center', va='center')

plt.xlabel("Models")

plt.ylabel("Mean AUC")

plt.title(f"Mean AUC Scores with {n_splits}-Fold Stratified Cross-Validation")

plt.xticks(rotation=45, ha='right')

plt.tight_layout()

plt.show()

# t-test

from scipy.stats import ttest_ind

model_names = list(results.keys())

p_values = pd.DataFrame(index=model_names, columns=model_names)

for i, model1 in enumerate(model_names):

for j, model2 in enumerate(model_names):

if i < j:

t_stat, p_value = ttest_ind(results[model1], results[model2])

p_values.loc[model1, model2] = p_value

p_values.loc[model2, model1] = p_value

# ANOVA

anova_f_stat, anova_p_value = f_oneway(*[results[model] for model in results.keys()])

# Visualization of p-values

plt.figure(figsize=(12, 10))

plt.imshow(p_values.astype(float), cmap='coolwarm', interpolation='nearest')

plt.colorbar(label="p-value")

plt.xticks(ticks=np.arange(len(model_names)), labels=model_names, rotation=45, ha='right')

plt.yticks(ticks=np.arange(len(model_names)), labels=model_names)

# Write p-values on the heatmap

for i in range(len(model_names)):

for j in range(len(model_names)):

if not pd.isna(p_values.iloc[i, j]):

value = p_values.iloc[i, j]

color = "white" if value < 0.05 else "black"

plt.text(j, i, f'{value:.3f}', ha='center', va='center', color=color)

# Set the title

plt.title("p-value Heatmap from t-tests")

plt.tight_layout()

plt.show()

# Display ANOVA results

print(f"ANOVA p-value: {anova_p_value:.3e}")

# Display p-value table

print("Pairwise t-test p-values:")

print(p_values)
