# Supplementary material for: Machine Learning–Based Prediction of Delirium and Risk Factor Identification in Intensive Care Unit Patients With Burns: Retrospective Observational Study
Source: JMIR Form Res. 2025 Mar 5;9:e65190. doi: 10.2196/65190 (PMC11923481; doi:10.2196/65190)
Supplement: Multimedia Appendix 5 [file formative_v9i1e65190_app5.docx]

# Load the data

import pandas as pd

data_path = '/content/drive'

df = pd.read_csv(data_path)

# Split features and target

X = df.drop("Delirium", axis=1) # Use "Delirium" column as the target

y = df["Delirium"]

# Split the data

from sklearn.model_selection import train_test_split

X_train, X_test, y_train, y_test = train_test_split(X, y, test_size=0.2, random_state=42)

# Train an AdaBoost model

from sklearn.ensemble import AdaBoostClassifier

from sklearn.metrics import accuracy_score

# Define the AdaBoost model

adaboost_model = AdaBoostClassifier(n_estimators=100, random_state=42)

# Train the model

adaboost_model.fit(X_train, y_train)

# Predict and evaluate the AdaBoost model

y_pred_adaboost = adaboost_model.predict(X_test)

accuracy_adaboost = accuracy_score(y_test, y_pred_adaboost)

print(f"AdaBoost Model Accuracy: {accuracy_adaboost:.4f}")

# Get feature importance

feature_importances_adaboost = pd.DataFrame({

'Feature': X.columns,

'Importance': adaboost_model.feature_importances_

}).sort_values(by='Importance', ascending=False)

# Display the top 15 important features

print(feature_importances_adaboost.head(15))

# Visualize the feature importance

import matplotlib.pyplot as plt

# Set figure size

plt.figure(figsize=(12, 8))

# Draw bar chart

plt.barh(feature_importances_adaboost['Feature'][:15], feature_importances_adaboost['Importance'][:15])

# Set x-axis label, y-axis label, and title

plt.xlabel('Importance')

plt.ylabel('Feature')

plt.title('AdaBoost Feature Importance')

# Invert y-axis

plt.gca().invert_yaxis()

# Display importance values on the right side of each bar

for index, value in enumerate(feature_importances_adaboost['Importance'][:15]):

plt.text(value, index, f'{value:.4f}', va='center')

# Remove the right and top borders

ax = plt.gca() # Get current axis

ax.spines['right'].set_visible(False) # Remove right border

ax.spines['top'].set_visible(False) # Remove top border

# Show the graph

plt.show()
