# Supplementary material for: Machine Learning–Based Prediction of Delirium and Risk Factor Identification in Intensive Care Unit Patients With Burns: Retrospective Observational Study
Source: JMIR Form Res. 2025 Mar 5;9:e65190. doi: 10.2196/65190 (PMC11923481; doi:10.2196/65190)
Supplement: Multimedia Appendix 8 [file formative_v9i1e65190_app8.docx]

# ------------------------------

# Import necessary libraries

# ------------------------------

import pandas as pd

import matplotlib.pyplot as plt

from sklearn.model_selection import train_test_split

from sklearn.discriminant_analysis import LinearDiscriminantAnalysis

from sklearn.metrics import accuracy_score

# ------------------------------

# Load the data

# ------------------------------

data_path = '/content/drive'  # correct path

df = pd.read_csv(data_path)

# Split features and target

X = df.drop("Delirium", axis=1)  # Use "Delirium" column as the target

y = df["Delirium"]

# Split the data

X_train, X_test, y_train, y_test = train_test_split(X, y, test_size=0.2, random_state=42)

# ------------------------------

# Train an LDA model

# ------------------------------

lda_model = LinearDiscriminantAnalysis()

# Define and train the model

lda_model.fit(X_train, y_train)

# Predict and evaluate the LDA model

y_pred_lda = lda_model.predict(X_test)

accuracy_lda = accuracy_score(y_test, y_pred_lda)

print(f"LDA Model Accuracy: {accuracy_lda:.4f}")

# ------------------------------

# Calculate feature contribution for the LDA model

# ------------------------------

lda_coefficients = pd.DataFrame({

    'Feature': X.columns,

    'Coefficient': lda_model.coef_[0]

}).sort_values(by='Coefficient', ascending=False)

# Display the top 15 LDA model coefficients

print(lda_coefficients.head(15))

# ------------------------------

# Visualize the contribution of explanatory variables

# ------------------------------

plt.figure(figsize=(12, 8))

plt.barh(lda_coefficients['Feature'][:15], lda_coefficients['Coefficient'][:15])

plt.xlabel('Coefficient')

plt.ylabel('Feature')

plt.title('LDA')

plt.gca().invert_yaxis()

# Display coefficient values on the right side of each bar

for index, value in enumerate(lda_coefficients['Coefficient'][:15]):

    plt.text(value, index, f'{value:.4f}', va='center')

# Remove the right and top borders

ax = plt.gca()

ax.spines['right'].set_visible(False)

ax.spines['top'].set_visible(False)

# Show the graph

plt.show()
