# Supplementary material for: Machine Learning–Based Prediction of Delirium and Risk Factor Identification in Intensive Care Unit Patients With Burns: Retrospective Observational Study
Source: JMIR Form Res. 2025 Mar 5;9:e65190. doi: 10.2196/65190 (PMC11923481; doi:10.2196/65190)
Supplement: Multimedia Appendix 10 [file formative_v9i1e65190_app10.docx]

# Import necessary libraries

import shap

import matplotlib.pyplot as plt

import numpy as np

import pandas as pd

from sklearn.model_selection import train_test_split

from sklearn.linear_model import LogisticRegression

from sklearn.preprocessing import LabelEncoder

# Mount Google Drive (for Colab environment)

from google.colab import drive

drive.mount('/content/drive')

# Load the data

data_path = '/content/drive'

df = pd.read_csv(data_path)

# Split features and target

X = df.drop("Delirium", axis=1) # Use the "Delirium" column as the target

y = df["Delirium"]

# Encode categorical data

for col in X.select_dtypes(include=['object']).columns:

le = LabelEncoder()

X[col] = le.fit_transform(X[col])

# Split the data

X_train, X_test, y_train, y_test = train_test_split(X, y, test_size=0.2, random_state=42)

# Train the model

models = {

"Logistic Regression": LogisticRegression(random_state=42, max_iter=1000)

}

models["Logistic Regression"].fit(X_train, y_train)

# Get SHAP values

explainer_lr = shap.Explainer(models["Logistic Regression"], X_train)

shap_values_lr = explainer_lr(X_train)

# Custom plot function

def custom_summary_plot(shap_values, feature_names, model_name, top_n=15):

# If the number of features is smaller than top_n, set top_n to the number of features

top_n = min(top_n, len(feature_names))

# Calculate mean absolute SHAP values

mean_abs_shap = np.mean(np.abs(shap_values.values), axis=0)

# Get top features

indices = np.argsort(mean_abs_shap)[-top_n:]

# Plot

plt.figure(figsize=(12, 8))

plt.title(f"{model_name}")

plt.barh(range(top_n), mean_abs_shap[indices])

plt.yticks(range(top_n), feature_names[indices])

plt.ylabel("Explanatory variables") # Set y-axis label

for i, v in enumerate(mean_abs_shap[indices]):

plt.text(v + 0.02 * max(mean_abs_shap[indices]), i, f"{v:.4f}", va="center", ha="left")

plt.gca().spines["top"].set_visible(False)

plt.gca().spines["right"].set_visible(False)

plt.xlabel("Mean absolute SHAP value")

plt.show()

# Display the summary plot

feature_names = np.array(X_train.columns)

shap_values_to_plot = shap_values_lr

custom_summary_plot(shap_values_to_plot, feature_names, "Logistic Regression")
