# Supplementary material for: Machine Learning–Based Prediction of Delirium and Risk Factor Identification in Intensive Care Unit Patients With Burns: Retrospective Observational Study
Source: JMIR Form Res. 2025 Mar 5;9:e65190. doi: 10.2196/65190 (PMC11923481; doi:10.2196/65190)
Supplement: Multimedia Appendix 11 [file formative_v9i1e65190_app11.docx]

# Load the data

import pandas as pd

data_path = '/content/drive'

df = pd.read_csv(data_path)

# Split features and target

X = df.drop("Delirium", axis=1) # Use "Delirium" column as the target

y = df["Delirium"]

# Split the data

from sklearn.model_selection import train_test_split

X_train, X_test, y_train, y_test = train_test_split(X, y, test_size=0.2, random_state=42)

# Train a Naive Bayes model

from sklearn.naive_bayes import GaussianNB

from sklearn.metrics import accuracy_score

# Define the Naive Bayes model

nb_model = GaussianNB()

# Train the model

nb_model.fit(X_train, y_train)

# Predict and evaluate the Naive Bayes model

y_pred_nb = nb_model.predict(X_test)

accuracy_nb = accuracy_score(y_test, y_pred_nb)

print(f"Naive Bayes Model Accuracy: {accuracy_nb:.4f}")

# Calculate feature importance using mean differences

feature_means = X_train.groupby(y_train).mean()

feature_diffs = abs(feature_means.loc[1] - feature_means.loc[0])

# Display the top 15 important features

feature_importances_nb = feature_diffs.sort_values(ascending=False).head(15)

print(feature_importances_nb)

# Visualize the feature importance

import matplotlib.pyplot as plt

# Set figure size

plt.figure(figsize=(12, 8))

# Draw bar chart

plt.barh(feature_importances_nb.index, feature_importances_nb.values)

# Set x-axis label, y-axis label, and title

plt.xlabel('Mean Difference')

plt.ylabel('Feature')

plt.title('Naive Bayes')

# Invert y-axis

plt.gca().invert_yaxis()

# Display importance values on the right side of each bar

for index, value in enumerate(feature_importances_nb.values):

plt.text(value, index, f'{value:.4f}', va='center')

# Remove the right and top borders

ax = plt.gca() # Get current axis

ax.spines['right'].set_visible(False) # Remove right border

ax.spines['top'].set_visible(False) # Remove top border

# Show the graph

plt.show()
