# Supplementary material for: Machine Learning–Based Prediction of Delirium and Risk Factor Identification in Intensive Care Unit Patients With Burns: Retrospective Observational Study
Source: JMIR Form Res. 2025 Mar 5;9:e65190. doi: 10.2196/65190 (PMC11923481; doi:10.2196/65190)
Supplement: Multimedia Appendix 12 [file formative_v9i1e65190_app12.docx]

# Load the data

import pandas as pd

data_path = '/content/drive'

df = pd.read_csv(data_path)

# Split features and target

X = df.drop("Delirium", axis=1) # Use "Delirium" column as the target

y = df["Delirium"]

# Split the data

from sklearn.model_selection import train_test_split

X_train, X_test, y_train, y_test = train_test_split(X, y, test_size=0.2, random_state=42)

# Build and train a Neural Network

from sklearn.neural_network import MLPClassifier

from sklearn.metrics import accuracy_score

# Define the Neural Network model

nn_model = MLPClassifier(hidden_layer_sizes=(100, 50), max_iter=500, random_state=42)

# Train the model

nn_model.fit(X_train, y_train)

# Predict and evaluate the Neural Network model

y_pred_nn = nn_model.predict(X_test)

accuracy_nn = accuracy_score(y_test, y_pred_nn)

print(f"Neural Network Model Accuracy: {accuracy_nn:.4f}")

# Calculate feature importance for the Neural Network model

nn_importance = pd.DataFrame({

'Feature': X.columns,

'Importance': nn_model.coefs_[0].mean(axis=1)

}).sort_values(by='Importance', ascending=False)

# Display the top 15 important features

print(nn_importance.head(15))

# Visualize the contribution of explanatory variables for the Neural Network model

import matplotlib.pyplot as plt

# Set figure size

plt.figure(figsize=(12, 8))

# Draw bar chart

plt.barh(nn_importance['Feature'][:15], nn_importance['Importance'][:15])

# Set x-axis label, y-axis label, and title

plt.xlabel('Importance')

plt.ylabel('Feature')

plt.title('Neural Network')

# Invert y-axis

plt.gca().invert_yaxis()

# Display importance values on the right side of each bar

for index, value in enumerate(nn_importance['Importance'][:15]):

plt.text(value, index, f'{value:.4f}', va='center')

# Remove the right and top borders

ax = plt.gca() # Get current axis

ax.spines['right'].set_visible(False) # Remove right border

ax.spines['top'].set_visible(False) # Remove top border

# Show the graph

plt.show()
